# Institutional Needs

**Published:** 1913-05-31

**Authors:** 


### Institutional Needs.

### LEVESON'S INVALID FURNITURE.

The bath chairs, spinal carriages, and invalid furniture manufactured by Messrs. Leveson and Sons, of Bloomsbury, London, so much appreciated for their quality of design and workmanship, are admirably summarised in the firm's fifty-eighth annual catalogue. It is evident that great pains have been expended in illustrating and describing the numerous designs in the best possible way, and it is instructive to note the improvements which increased experience of the needs of the invalid, and the application to those needs of carefully thought out designs by competent artisans, can accomplish in securing the greatest amount of comfort at a price consistent with good material and workmanship. Such improvements are especially evidenced in the construction of the spinal carriage, in which accuracy of balance and lightness of draught are combined with a neatness of outline which contrasts favourably with the old-fashioned box-like carriage. A really good catalogue of this type has much value as a work of reference, and it is odd that more files are not kept of volumes of this class.

